# *Androctonus* genus species in arid regions: Ecological niche models, geographical distributions, and envenomation risk

**DOI:** 10.14202/vetworld.2018.286-292

**Published:** 2018-03-06

**Authors:** Moulay Abdelmonaim El Hidan, Oulaid Touloun, Abdellah Bouazza, Mehdi Ait Laaradia, Ali Boumezzough

**Affiliations:** 1 Department of Biology, Laboratory of Ecology and Environment, Faculty of Sciences Semlalia, Cadi Ayyad University, Marrakesh 40000, PO Box 2390, Morocco; 2 Department of Biology, Polyvalent Laboratory of Research & Development LPVRD, Polydisciplinary Faculty, Sultan Moulay Slimane University, Beni Mellal, PO Box 23023, Morocco; 3 Faculty of Sciences, Biodiversity and Ecosystem Dynamics Laboratory, B.P. 2390, Cadi Ayyad University, Marrakech 40000, Morocco; 4 Department of Biology, Laboratory of Pharmacology, Neurobiology and Behavior, Faculty of Sciences Semlalia, University Cadi Ayyad, Marrakesh, Morocco

**Keywords:** *Androctonus* genus, ecological niche models, Morocco, risk maps, scorpion envenomation

## Abstract

**Aim:**

The objective of this study was to establish environmental factors related to scorpion species occurrence and their current potential geographic distributions in Morocco, to produce a current envenomation risk map and also to assess the human population at risk of envenomation.

**Materials and Methods:**

In this study, 71 georeferenced points for all scorpion species and nine environmental indicators were used to generate species distribution models in Maxent (maximum entropy modeling of species geographic distributions) version 3.3.3k. The models were evaluated by the area under the curve (AUC), using the omission error and the binomial probability. With the data generated by Maxent, distribution and envenomation risk maps were produced using the “ESRI^®^ ArcGIS 10.2.2 for Desktop” software.

**Results:**

The models had high predictive success (AUC >0.95±0.025). Altitude, slope and five bioclimatic attributes were found to play a significant role in determining *Androctonus* scorpion species distribution. Ecological niche models (ENMs) showed high concordance with the known distribution of the species. Produced risk map identified broad risk areas for *Androctonus* scorpion envenomation, extending along Marrakech-Tensift-Al Haouz, Souss-Massa-Draa, and some areas of Doukkala-Abda and Oriental regions.

**Conclusion:**

Considering these findings ENMs could be useful to afford important information on distributions of medically important scorpion species as well as producing scorpion envenomation risk maps.

## Introduction

Scorpion envenomation is an important public health problem in many parts of the world; Central and South America, North Africa, the Middle East, and South Asia. Most venomous scorpions belong to the Buthidae family, which comprises species from the genera *Androctonus* and *Buthus* in North Africa, *Tityus* in South America, *Centruroides* in North and Central America, and *Mesobuthus* in Asia. The genus *Androctonus* is one of the largest and most widely distributed genera of the family Buthidae in North Africa. Species belonging to this genus are most frequently incriminated in severe scorpion stings. In fact, in the Maghreb area, scorpions of *Androctonus* genus are responsible for about 100,000 stings per year [1]. In Morocco, the genus *Androctonus* is represented by seven scorpion species [2].

Reporting of scorpion envenomation, by health authorities is generally deficient in most developing countries, as the majority of the patients first consulted traditional practitioners, which lead to a lack of information on scorpion stings and lethality incidence [3,4]. To evaluate accurate scorpion stings incidence and envenomation, it is important to count on community-based epidemiological studies independent of hospital reporting. However, this method would be expensive to apply at large scales [5]. Predictive models tools such as Geographic Information Systems (GIS) and ecological niche modeling (ENM) have been used to identify high envenomation risk regions related to other venomous animals (snakes and spiders) [6-8]. ENMs combined with GIS allow identifying high vulnerability regions to venomous animal and predicting species geographic responses to environmental variables as well. Whereas it takes into consideration the individual ecologies of those animals, and as a consequence, it gives unique opportunity to produce a risk map with the full complexity of the phenomenon.

Therefore, we used ENM and GIS techniques to establish environmental factors related to species occurrence and current potential geographic distributions for species belonging to the *Androctonus* genus in Morocco, to produce a current envenomation risk map and also assessing the human population at risk of envenomation with species of this genus.

## Materials and Methods

### Ethical approval

This study was subject to the regulations of Public and Ethics Health committee in Morocco.

### Study area

Morocco is located on the westernmost tip of North Africa with a surface of 720,000 km^2^. Characterized by its geography includes no <4 separate mountain ranges and wide expanses of desert. The three most prominent mountain ranges, which run parallel to each other from the southwest to the northeast, are the Middle Atlas, the High Atlas, and the Anti-Atlas. Moreover, the country is bordered by the North Atlantic Ocean to the west, and the west Mediterranean Sea to the north . In the south of the country, the Sahara is the largest desert in the world.

The climate in Morocco is as varied as its diverse geography. In general, the country has a Mediterranean climate. The coast has a warm, Mediterranean climate tempered on the eastern coast by southwest trade winds while inland areas have a hotter, drier, and continental climate. In the south of the country, the weather is very hot and dry throughout most of the year, though temperatures can drop dramatically at night, especially in the months of December-January.

### Study species

Seven species’ belong to *Androctonus* genus are present in Morocco: *Androctonus amoreuxi*, *Androctonus australis*, *Androctonus gonneti*, *Androctonus liouvillei*, *Androctonus mauritanicus*, *Androctonus maroccanus*, and *Androctonus sergenti*. However, we removed rare species since the meeting probability of these scorpions with humans is low, and because presence records for these species are scarce, which affects the performance of ENMs [6,9]. Thus, we had chosen as study species only species with six or more presence data in the country: *A. amoreuxi*, *A. liouvillei*, and *A. mauritanicus*.

### Scorpion sampling and identification and species range recording

To locate scorpions, the ground was examined by lifting rocks, stones, and tree bark. The burrows considered to be occupied by scorpions were destroyed with a shovel to try to dislodge them. For the anthropophilic species, we investigated under stones and near indoor dwellings. The property that renders the scorpion carapace strongly fluorescent under ultraviolet light creates an excellent opportunity to detect these nocturnal arachnids. Hence, the nocturnal missions were conducted, using portable ultraviolet lamps. Specimens were identified in the laboratory using the keys and descriptions published by Vachon and Lourenço [2,10,11].

Distributional data representing 71 records (i.e., unique species × latitude-longitude combinations) for the three *Androctonus* species (*A. amoreuxi* - 22, *A. liouvillei* - 18, and *A. mauritanicus* - 31) were used to develop models, all of the records were unpublished observations by the authors. Observations were collected from the period between 2005 and 2015. For all observations, the geographic location was recorded with a Global Positioning System.

### Environmental data

Environmental data used for species distribution models were represented by bioclimatic layers of current climatic trends [12]. To improve model precision and decrease problems with extrapolation, layers were clipped to the ecoregions that comprised occurrence records [13]. To avoid overfitting the models and improve model transferability, Pearson correlations between layers were calculated using ENMTool v. 1.3 [14]. When the Pearson’s correlation coefficient was >0.75 [15] amid layers, one of the layers was chosen for models construction. These layers included in addition to the altitude and slope (derived from altitude raster with the “Slope” function of ArcGIS), seven climate grids (Table-1) [12].

**Table-1:** Environmental factors used for model the distribution of Scorpion in Morocco and their codes and units.

Code	Variable	Unit
ALT	Altitude	M
SLOP	Slope	%
Bio-2	Mean diurnal range	°C
Bio-9	Mean temperature of driest quarter	°C
Bio-16	Precipitation of wettest quarter	Mm
Bio-18	Precipitation of warmest quarter	Mm
Bio-19	Precipitation of coldest quarter	Mm

### Ecological niche-based models

Models were constructed with the maximum entropy approach [16]. This modeling technique requires only presence data as input, and consistently performed well in comparison to other methods [17], especially at low samples sizes [9,18], scorpion records and environmental data were imported into MaxEnt v. 3.3.3 software [16]. A total of 20 model replicates were run with random seed. Thus, 20% of the presence points were used as test points and 80% for model training [19]. Records for each replicate were selected by bootstrap permitting sampling with replacement. Models were run with auto-features [16]. Model performance was assessed by the default evaluation of the area under the receiver operating characteristic curve (AUC) [20].

### Human population projections for risk assessment

Human population data for the year 2015 were obtained from the Gridded Population of the World, Version 4 (GPWv4) [21]. The distribution layers had a resolution of 1 km. These layers were overlaid with the models that predicted the presence of at least one scorpion species. The human population potentially exposed to scorpion envenomation was computed as that of those cells in which at least one scorpion species was present at the 10% levels.

### Envenomation risk maps

To predict the probability of being envenomed by one of *Androctonus* scorpion species over the study area, we developed a risk map using the Maxent models of the three species. Performing risk map involves four steps:

- Map (1): Binary maps for each species were produced using the minimum presence value threshold value [6]. Before stacking and summing these layers, each species was weighted by its biological features linked to scorpion envenomation risk (see supplementary material).
- Map (2): Environmental suitability maps were developed from the seven components used in model calibration for all cells identified as suitable by the binary models. Briefly, maps of various environmental components were reclassified, and every class was weighted according to the probability of occurrence of each species. Then for each species, we overlaid and summed the seven environmental layers multiplying each by their weight which corresponds to their percent contribution. Finally, the three resulted layers were also overlaid and summed.
- Map (3): Most of the studies used population distribution and density to describe and model the risk of disease occurrence. It was presumed that scorpion envenomation incidence is related to the probability of encountering between human and scorpions and this probability is higher when the population density is high. Based on this assumption, map risk was computed using also a population density map derived from GPWv4 [21], this map was reclassified to 10 classes, and each class was weighted from 1 to 10 depending on the population density.

All the three resulted maps were stacked, summed, and normalized to lie between 0 and 1 which was interpreted as a relative measure of expected envenomation risk rate.

## Results

ENMs produced in this study were better than random, as the ROC plots exhibited high average AUCs with low standard deviations for both training and test datasets in all models (Table-2).

**Table-2:** AUC and SD values obtained from internal and external model evaluation.

Model performance	AUC for training data	AUC for test data	SD
*A. amoreuxi*	0.951	0.950	0.025
*A. liouvillei*	0.987	0.988	0.006
*A. mauritanicus*	0.975	0.969	0.007

Note that for AUC the specificity is defined using predicted area, rather than true commission. This implies that the maximum achievable AUC is <1. *A. amoreuxi*: *Androctonus amoreuxi*, *A. liouvillei*: *Androctonus liouvillei*, *A. mauritanicus*: *Androctonus mauritanicus*, AUC=Area under the curve, SD=Standard deviation

From the results of ecological models, we could distinguish ecological variables related to the distribution of each scorpion species. Thus, *A. amoreuxi* is mostly related to the mean temperature of driest quarter, mean diurnal range, and slope. While *A. liouvillei* is related to mean diurnal range, slope, precipitation of wettest quarter, and precipitation of warmest quarter. Finally, *A. mauritanicus* is related to precipitation of coldest quarter, altitude and precipitation of wettest quarter and precipitation of warmest quarter (Table-3).

**Table-3:** Response values percent contribution and permutation importance (in %) of the predictor variables for the ecological niche models of *A. amoreuxi, A. liouvillei*, and *A. mauritanicus* from Morocco.

Variables	Percent contribution			Permutation importance		
	
	*A. amoreuxi*	*A. liouvillei*	*A. mauritanicus*	*A. amoreuxi*	*A. liouvillei*	*A. mauritanicus*
Alt	9.6	7.4	12.2	12.4	24.3	19
Slop	20.7	18.6	4.6	20.7	37	2.5
Bio2	21.2	28.5	8.1	10.7	4.3	2.4
Bio9	28.1	6.4	0.5	2.3	0.2	0.9
Bio16	2.8	17.5	0.9	11.1	28.3	5.9
Bio18	4.4	17.1	10.1	4.3	5.4	3.6
Bio19	13.2	4.6	63.6	38.4	0.5	65.7

A. amoreuxi=Androctonus amoreuxi, A. liouvillei=Androctonus liouvillei, A. mauritanicus=Androctonus mauritanicus

**Figure-1:**
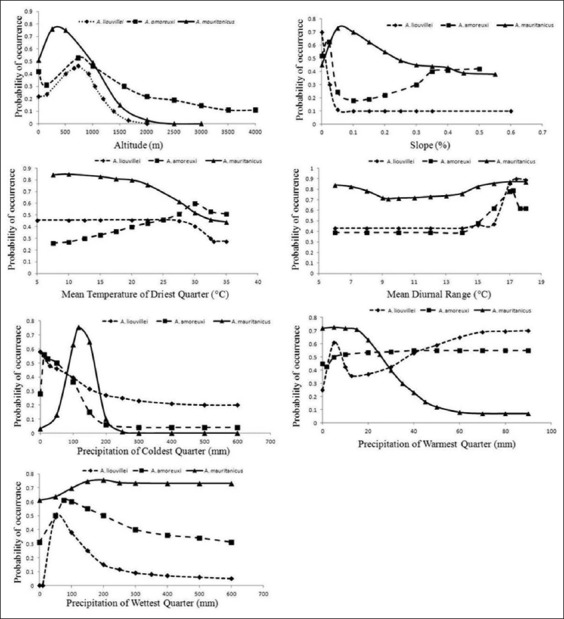
Response curves for the most related environmental factors to the distribution of *Androctonus* scorpion species in Morocco. Curves depict the average probability of occurrence from 20 model replicates along with the environmental gradients.

The response of the three scorpion species toward each ecological factor is appraised based on response curves (Figure-1). All three species occur more frequently in areas with an altitude between 0 and 1500 m maximum density was observed at an approximate elevation of 700 m for *A. liouvillei* and *A. amoreuxi*, while for *A. mauritanicus* it was at 450 m.

The three scorpion species respond differently to temperature variables (Figure-1), in fact, *A. mauritanicus* occur more frequently in areas with mean temperature of driest quarter between 6 and 25°C and between 6 and 19°C for mean diurnal range, while *A. liouvillei* is more likely present between 5 and 30°C for mean temperature of driest quarter and between 16 and 18°C for mean diurnal range. The probability of the presence of *A. amoreuxi* is elevated in areas when mean temperature of driest quarter and mean diurnal temperature are ranged between 25-35°C and 15-19°C, respectively.

Precipitation is another factor that affects the distribution of three scorpion species. In general, all three species occur in regions with low precipitation of coldest quarter (0-200 mm). Regarding precipitation of warmest quarter, *A. amoreuxi* had a maximum probability of presence of 0.5 between 10 and 80 mm, while *A. mauritanicus* occur mainly in areas with low precipitation of warmest quarter (0-30 mm) in contrast to *A. liouvillei* which is principally present in regions with high precipitation of warmest quarter (60-90 mm). For the last factor of precipitation, *A. liouvillei* and *A. amoreuxi* occur mainly in areas with low precipitation of wettest quarter with a maximum occurrence probability at 80 and 100 mm, respectively (Figure-1).

**Figure-2:**
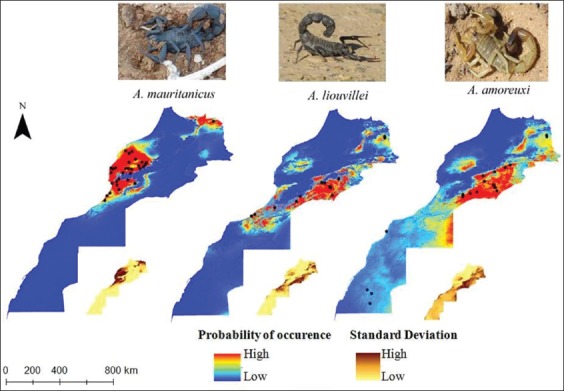
Average and standard deviation (small insets) of probability of occurrence of *Androctonus* scorpion species at a 1*1 km scale estimated from the ensemble of 20 model replicates.

Ecological models allowed identifying suitable habitats for the occurrence of the three scorpion species. Appropriate areas for *A. amoreuxi* were predicted mostly along parts of the Moroccan east from Mediterranean coast until Semara. Areas of high occurrence probability for *A. liouvillei* were located also in the east of the country as well as at some areas along the Atlantic coastline from Agadir to about Akhfennir. High presence probability for *A. mauritanicus* is located along Atlantic coastline between Eljadida and Tiznit as at some area in the northeast part of the study area (Figure-2).

**Figure-3:**
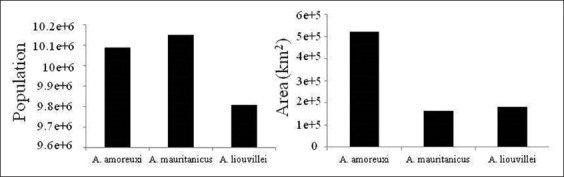
Histograms showing the area and human population under scorpion envenomation risk according to scorpion species.

Distribution area indicates a high occupied surface by *A. amoreuxi* flowed by *A. liouvillei* and *A. mauritanicus*. However, the number of people at risk of envenomation by *A. mauritanicus* is higher than the two other species (Figure-3).

**Figure-4:**
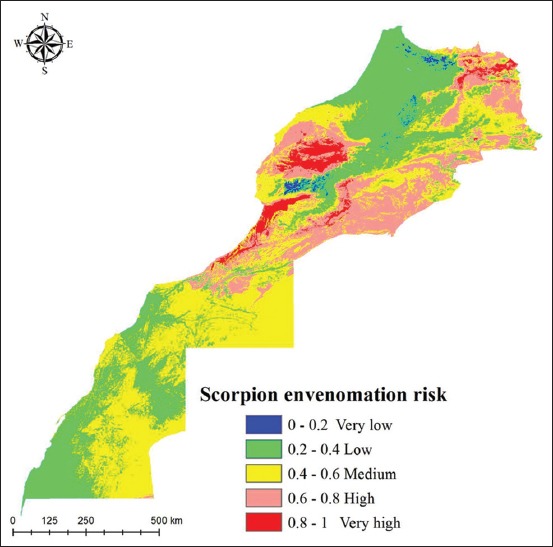
*Androctonus* scorpion envenomation risk predictions in Morocco.

Scorpion envenomation risk map shows a well-spread risk through Morocco with “very high risk” regions located mainly in Marrakech-Tensift-Al Haouz (MTH), Souss-Massa-Draa (SMD), and some areas of Doukkala-Abda (DA), and Oriental regions. “High risk” also was situated in the same regions although limited parts in Tadla-Azilal (TA), Chaouia-Ouardigha (CO), Fes-Boulmane, Meknes-Tafilalet, and Guelmim-Es-Semara also fell in this class. The other categories with “Medium, very low, and low envenomation risk” are principally in the North-West regions and southern regions of the country (Figure-4).

## Discussion

ENM has been extensively used to predict the distribution of species related to diseases transmission [22].

The use of niche model tools could help for the characterization of medically important scorpion species distribution, and eventually predict the spatial risk of their suitable habitats. This may facilitate performing risk assessment maps and conception of specific control measures. GIS/ENM tools were successfully used in mapping envenomation risk for other venomous animals such as snakes and spiders [6-8,23]. The present study is the first attempt to use GIS/ENM to model current spatial distribution and scorpion envenomation risk in Morocco.

Environmental variables, such as topography and climate, can play important roles in envenomation extent by affecting the distribution and abundance of venomous animals. Indeed climate could affect scorpion distribution by two main components; rainfall (relative humidity) and temperature [24]. In fact, Warburg *et al*. had reported that rainfall has an obvious effect on scorpion distribution in Israel [25], while temperature constitutes the main factor limiting the southward expansion of tropical scorpion species on the east coast of Australia [26]. In this study, all environmental variables appear to contribute positively to the final predictive models. However, we found that each species was most strongly associated with specific environmental factors. Thus mean diurnal range, slope precipitation of wettest quarter, and warmest quarter were consistently related to the distribution of *A. liouvillei*. Whereas *A. amoreuxi* was more associated to the mean temperature of driest quarter, mean diurnal range, and slope while *A. mauritanicus* was mostly affected by precipitation of coldest and warmest quarter.

Scorpion envenomation risk maps were based on ENMs produced for a medically important scorpion genus (*Androctonus*) in Morocco. Based on our evaluations, all niche models were well performed. We produced detailed current distributional maps for the three species most medically relevant and the most widespread in Morocco.

Our results reflect the known distribution of the three scorpion species fairly well. However, modeled distribution of *A. amoreuxi* and *A. liouvillei* predicts the occurrence of the two species in the region of Marrakech, even if the presence of these species had never been mentioned in. This incongruence either indicates model failure, with models including regions not ecologically suitable for the species, or the models are correct, and the two species are not found in Marrakech region due to the presence of barriers which limited dispersion potential. The latter scenario is more plausible with the presence of large geographical barrier represented in the Atlas Mountains which is known to limit the dispersion of arthropods especially scorpions [27].

*Androctonus* is possibly the most dangerous genus in North Africa, because of its wide distribution, size, and the high toxicity of its venom [28]; also species belonging to this genus is well adapted to anthropized habitats, making scorpion-human encounters frequent especially close to rural dwellings.

Current scorpion envenomation risk predictions show a high envenomation risk in MTH, SMD, and some parts of DA, those results corroborate previous data. In fact, Aboumaâd *et al*. [29] in his review on scorpion envenomation in Morocco had reported that the three regions with high envenomation incidence are orderly MTH, SMD, and CO. Furthermore, epidemiological study conducted in MTH and SMD had reported that scorpion envenomation is an important health problem in those two regions with a high incidence and lethality rate [30].

In spite of our focus on species belonging to the genus *Androctonus* as a principal cause of scorpion envenomation in Morocco, we note that the situation must be considerably more complex in reality. That is, considering the distribution of this species, it cannot account for the full envenomation incidence as regions of known “high envenomation incidence” such as CO and TA fall outside of the “high risk” area. This could be explained by the presence of other medically important species characterized by their highly toxic venom and wide distribution such as *Hottentotta gentili* and *Buthus occitanus* [31].

## Conclusion

This study demonstrates the ability of GIS/ENMs to supply detailed information on potential distributions of a medically important scorpion, in addition to estimating and mapping scorpion envenomation risk. Finally, risk maps could help public health authorities decide where to perform scorpion envenomation surveillance and take preventive measures.

## Authors’ Contributions

Data were collected and interpreted by MAE, OT, and AB using MaxEnt and ArcGIS. The manuscript was prepared jointly by MAE and MAL. AB participated in the review process and incorporated valuable suggestions for improvement of the manuscript. All authors read and approved the final manuscript.
